# Study of Nutrition, Health, and Food Security of Indigenous Peoples in the State of Alagoas, Brazil (ENSSAIA): Methodological Aspects

**DOI:** 10.1002/fsn3.71361

**Published:** 2025-12-25

**Authors:** Tamara Rodrigues dos Santos, Valéria Clarisse de Oliveira, Marly Augusto Cardoso, Monica Lopes de Assunção, Andreia Herculano da Silva, Regina Coeli da Silva Vieira, Lídia Bezerra Barbosa, Willams Henrique da Costa Maynart, Ewerton Amorim dos Santos, Haroldo da Silva Ferreira

**Affiliations:** ^1^ Faculty of Nutrition Federal University of Alagoas Maceió AL Brazil; ^2^ Graduate Program in Public Health, School of Public Health University of São Paulo São Paulo SP Brazil; ^3^ Department of Nutrition, School of Public Health University of São Paulo São Paulo SP Brazil; ^4^ Institute of Biological Sciences and Health Federal University of Alagoas Maceió AL Brazil; ^5^ Institute of Health and Biotechnology Federal University of Amazonas Coari AM Brazil; ^6^ School of Nursing Federal University of Alagoas Maceió AL Brazil; ^7^ Faculty of Medicine Federal University of Alagoas Maceió AL Brazil

**Keywords:** epidemiology, ethnic origin and health, health vulnerability, indigenous population, methods

## Abstract

This article describes the methodological aspects of the Study of Nutrition, Health, and Food Security of Indigenous Peoples in Alagoas (ENSSAIA), conducted between August 2022 and December 2023. The objective of ENSSAIA was to investigate the nutrition, health, and food security conditions of indigenous peoples in Alagoas. This was a cross‐sectional, population‐based household survey assessing all 11 indigenous ethnic groups in the state, with a random selection of 14 out of 29 existing villages. All individuals registered in the Indigenous Health Care Subsystem of the selected villages were eligible for the study. A total of 1400 families were assessed, with data collected on environmental, demographic, and socioeconomic conditions; access to policies and public service infrastructure; household food security; health of children (< 5 years), pregnant women, women (19–59 years), and the elderly (≥ 60 years); breastfeeding practices among children < 2 years; individuals with disabilities and genetic alterations; nutritional status through anthropometric indicators (all household members), biochemical markers: hemoglobin (children aged 6 months to < 5 years and pregnant women), lipid profile, and glycated hemoglobin (women); dietary indicators (women, pregnant women, and children < 5 years); non‐communicable diseases; and alcohol and tobacco use (women, men, and elderly). The results produced by ENSSAIA are expected to support the formulation or revision of public policies that ensure greater access to healthcare, healthy food, and improved quality of life for indigenous peoples, considering their traditions, cultures, and ways of life.

## Graphical abstract

The ENSSAIA Study is the first population‐based investigation encompassing all Indigenous ethnic groups in the state of Alagoas, Brazil. By integrating socioeconomic, dietary, nutritional, biochemical, and health indicators, the study provides robust evidence to support culturally contextualized health promotion interventions. Its methodological design ensures internal validity and comparability, offering a strategic framework for long‐term monitoring of food security and noncommunicable diseases among vulnerable Indigenous communities.

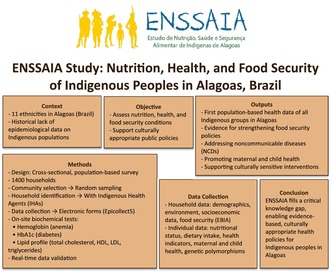

**Abbreviations**
24HR: 24‐Hour Dietary Recall
ASSIST: Alcohol, Smoking, and Substance Involvement Screening Test
BPM: Blood Pressure Measurement
CONDISI: District Indigenous Health Council
CONSEA/AL: State Council for Food and Nutrition Security of Alagoas
DSEI: Special Indigenous Health District of Alagoas and Sergipe
EBIA: Brazilian Food Insecurity Scale
EBIA‐G: Brazilian Food Insecurity Scale proposed and validated a version for use among the Guarani indigenous population of São Paulo's coastal region
ENSSAIA: Nutrition, Health, and Food Security Study of Indigenous Peoples in the State of Alagoas
FS: Food security status
GPS: geographical location
IHA: Indigenous Health Agent
PNSF: National Iron Supplementation Program
PNSVA: National Vitamin A Supplementation Program
SasiSUS: Indigenous Health Care Subsystem
SRQ‐20: Self‐Report Questionnaire
UFAL: Federal University of Alagoas
USP: University of São Paulo
VES‐13: Vulnerable Elders Survey‐13
Vigitel: Surveillance System for Risk and Protective Factors for Chronic Diseases by Telephone Survey
WHO: World Health Organization

## 1 Introduction

In Brazil, Indigenous populations have faced genocide and political, economic, and social exclusion since colonial times, a process that continues today. Despite legal protections established after the 1988 Federal Constitution, they consistently exhibit worse socioeconomic and health indicators than the general population. This heightened vulnerability threatens their cultural diversity and quality of life (Zelic et al. 2021; Campos et al. 2017; Marinho et al. 2019; Instituto Brasileiro de Geografia e Estatística 2024).

Indigenous peoples make up about 1% of Brazil's population, with the Northeast having the second‐largest Indigenous population. In Alagoas—the country's second smallest state and one of the most socioeconomically vulnerable—11 ethnic groups live across 29 communities. Proportionally, the state ranks 13th in Indigenous population, yet no prior representative data on their nutrition, health, and food security were available (Secretaria de Estado do Planejamento Gestão e Patrimônio 2017; Instituto Brasileiro de Geografia e Estatística 2023).

In this context, researchers from the Federal University of Alagoas (UFAL) have been conducting surveys with representative samples of populations in different settings since 2005 (Alagoas—2005 and 2015; semi‐arid region—2007; Quilombola population—2008 and 2018; northern region—2010; and schoolchildren in Maceió—2013).

At a December 2017 meeting of the Alagoas Food and Nutritional Security Council (CONSEA), where survey results were presented, Indigenous leaders questioned the absence of data on their population. After confirming the lack of epidemiological studies on Indigenous peoples in Alagoas, researchers from UFAL, and these leaders committed to developing a project to address this gap.

The Nutrition, Health, and Food Security Study of Indigenous Peoples in the State of Alagoas (ENSSAIA) reflects this commitment and was designed to investigate the nutritional, health, and food security conditions of these peoples, as well as to identify possible associated factors. This paper aims to disclose the methodological aspects of ENSSAIA.

Despite advances in the production of epidemiological data on the Brazilian population over recent decades, Indigenous peoples have historically been excluded from national health, nutrition, and food security surveys. The lack of representative and disaggregated data on these populations not only perpetuates their invisibility but also hampers the formulation, implementation, and monitoring of public policies aligned with their specific needs. The ENSSAIA study was designed precisely to address this gap, providing the first population‐based survey to include all Indigenous ethnic groups in the state of Alagoas. The methodological approach adopted involved important challenges and innovations, particularly in ensuring cultural appropriateness, respecting traditional knowledge, and adapting validated instruments to the Indigenous context. Describing these methodological aspects in detail is crucial not only to ensure transparency and reproducibility but also to serve as a reference for researchers, health professionals, and policymakers interested in developing similar studies with Indigenous peoples or other socio‐culturally diverse and vulnerable populations. By sharing the processes, adaptations, and lessons learned, this manuscript aims to contribute to the strengthening of epidemiological research focused on equity and cultural sensitivity, fostering the production of comparable, high‐quality data to support health surveillance and public policy development.

## 2 Methods

ENSSAIA is a cross‐sectional, population‐based household survey that assessed a probabilistic representative sample of the Indigenous peoples of Alagoas, most of whom are distributed between the Agreste and Sertão regions of the state (Table S1). All households in the selected communities with individuals registered in the Indigenous Healthcare Subsystem (SasiSUS) were eligible for the study. SasiSUS is a network of health actions and services within Brazil's Unified Health System (SUS) specifically designed for Indigenous populations.

Although the project's primary outcome is food insecurity, a conservative prevalence of 50% was adopted in the sample planning due to its broad survey scope. This choice aimed at maximizing the sample size, ensuring robust estimates for all outcomes. By maximizing variability, this approach enhances precision and accuracy, regardless of the actual prevalence of the outcomes investigated (Lwanga et al. 1991). In umbrella surveys, this strategy is especially relevant to ensure statistical validity and generalization of results (Kotrlik and Higgins 2001).

Thus, considering an estimated total of approximately 4036 families (based on the total number of Indigenous people registered in SasiSUS in 2022, divided by the average of three people per household according to the 2022 Census) (Instituto Brasileiro de Geografia e Estatística 2023; Brasil 2022), a sampling error of 2.5%, and a 95% confidence interval, a sample of 1113 families was required. It should be noted that for the outcome “food insecurity,” the unit of analysis is the family; however, for some other outcomes, the unit of analysis is the individual.

In Alagoas, there are 11 Indigenous ethnic groups distributed across 29 communities (Secretaria de Estado do Planejamento Gestão e Patrimônio 2017; Brasil 2022). While some ethnic groups are concentrated in a single community, others occupy multiple locations. To ensure greater representativeness and data diversity, the sample included families from one community per ethnic group, resulting in the selection of 11 communities. For ethnic groups with only one community (Aconã, Kariri‐Xocó, Katokinn, and Wassú), these were automatically included with a probability of 1 (Secretaria de Estado do Planejamento Gestão e Patrimônio 2017). For the remaining ethnic groups with multiple communities, a simple random sampling procedure was applied to select one community per group. This was done using the randbetween() function in Microsoft Excel, which generates random numbers to ensure unbiased selection.

Given the defined sample size (*n* = 1113 families; ≈3339 people) and the need for ethnic group balance, sub‐samples were required in communities with over 1500 inhabitants, such as Kariri‐Xocó and Wassú‐Cocal. Instead of including the entire population, randomly selected sectors covering about one‐third of residents were chosen. On the other hand, when a selected community comprised less than one‐third of the total ethnic population, additional areas from other communities of the same ethnic group were included. As a result, two communities had their samples supplemented by residents from nearby areas. In the Koiunpanká ethnic group, Baixa Fresca was supplemented by families from Roçado and Baixa do Galo, while in the Karuazu ethnic group, Tanque was supplemented by families from Campinhos. Although individuals from 14 locations were surveyed, Roçado, Baixa do Galo, and Campinhos were considered complementary to the 11 originally selected communities.

In both cases, these sectors and complementary areas were registered with SasiSUS and served by an Indigenous Health Agent (IHA), whose areas of responsibility are defined based on demographic, geographic, ethnic, cultural, and access criteria, as well as on the organization of services within the communities. Thus, to ensure the necessary sample, a simple random sampling was carried out among the IHA of each community until the target number of participants was reached. The communities selected according to the criteria described are presented in Table 1.

**TABLE 1:** Ethnic groups and respective communities investigated in ENSSAIA, Alagoas, 2023.

Region	City	Ethnicity	Communities
Agreste	Traipu	Aconã	Aconã
	São Sebastião	Karapotó	Fazenda Terra Nova
	Campo Grande, Feira Grande	Tingui‐Botó	Tingui‐Botó
Alto Sertão	Água Branca	Kalankó	Lajedo do Couro
	Inhapi	Koiunpanká	Baixa Fresca/Roçado^a^/Baixa do Galo^a^
	Pariconha	Geripankó	Serra do Engenho
	Pariconha	Karuazu	Tanque/Campinhos^a^
	Pariconha	Katokinn	Katokinn
Baixo São Francisco	São Brás, Porto Real do Colégio	Kariri‐Xokó	Kariri‐Xokó^b^
Planalto da Borborema	Palmeira dos Índios	Xucuru‐Kariri	Mata da Cafurna
Serra dos Quilombos	Colônia Leopoldina, Joaquim Gomes, Matriz de Camaragibe, Novo Lino	Wassú	Wassú‐Cocal^b^
Total		11	14

^a^ Community with sectors that supplemented the randomly selected community.

^b^ Community with more than 1500 people, in which sectors were randomly selected within the coverage area of an Indigenous Health Agent to cover approximately one third of the community population.

Household identification was carried out with the support and supervision of the IHA responsible for the respective area. In addition to identifying eligible households, particularly in locations where there are both Indigenous and non‐Indigenous households, the IHA played a crucial role in facilitating interactions between researchers and participants, acting when significant health problems were identified or arranging necessary referrals.

### 2.1 Data Collection

**FIGURE 1:**
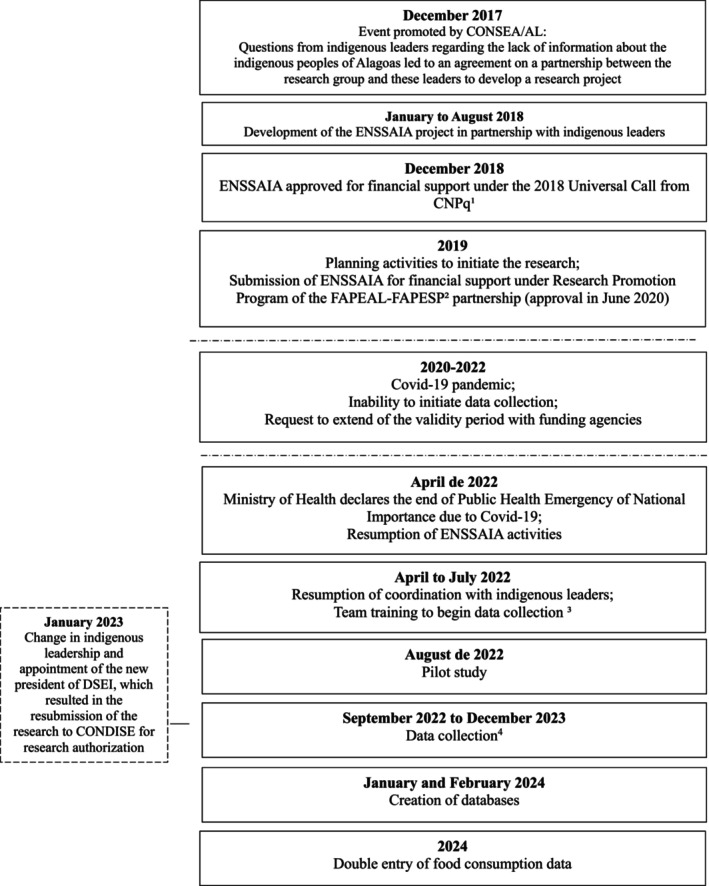
Research flowchart, from its conception to the completion of data collection. ENSSAIA, Alagoas, 2023. CONSEA/AL: State Council for Food and Nutrition Security of Alagoas. CNPq: National Council for Scientific and Technological Development. FAPEAL: Alagoas State Research Support Foundation. FAPESP: São Paulo State Research Support Foundation. DSEI: Special Indigenous Health District of Alagoas and Sergipe. CONDISE: District Indigenous Health Council. ^1^MCTIC/CNPq n° 28/2018—Universal; Process 432,249/2018‐4; ^2^Scientific Research Grant—FAPEAL/FAPESP; Process n° 600,300,000,000,460/2020; ^3^Master's and doctoral students who were part of the research when the project was approved in 2018 were no longer part of the group; ^4^February–March/2023: Data collection pause due to training of new team members (master's students) and June–July/2023: Data collection pause due to breakdown of the equipment used in the biochemical assessment.

**FIGURE 2:**
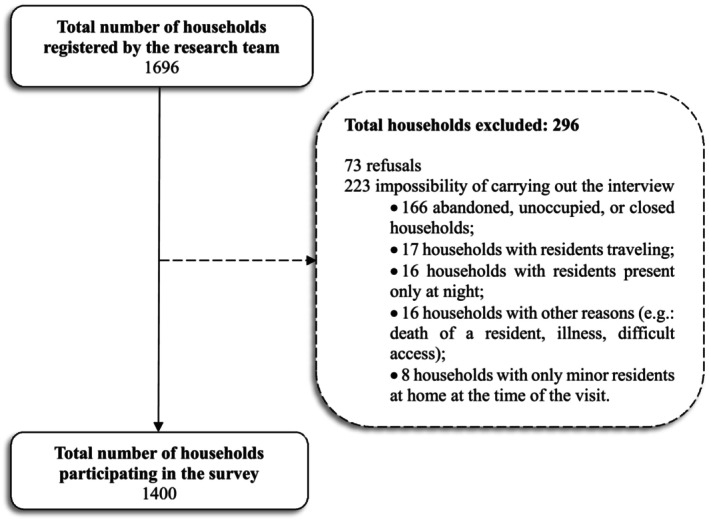
Flowchart of the total number of households identified, excluded, and evaluated. ENSSAIA, Alagoas, 2023.

Figure 1 presents the research flowchart, from its conception to the development of the databases. Data collection took place from September 5, 2022, to December 22, 2023. Of the 1696 registered households, 296 were excluded (Figure 2), resulting in 1400 households being evaluated.

Data collection was preceded by three stages: definition of instruments, team training, and a pilot study.

After defining the instruments, an interviewer manual was developed, detailing the responsibilities assigned to each role within the team and describing all procedures.

The field team consisted of 14 researchers (undergraduate and graduate students in health sciences) and a driver, with the following roles: one coordinator, one field supervisor, one anthropometrist, one laboratory assistant, and ten interviewers. Among the interviewers, two nutritionists were exclusively responsible for conducting dietary intake surveys. The coordination and supervision of field activities were led by a faculty member from UFAL and a postdoctoral researcher, both with extensive experience in household surveys.

**FIGURE 3:**
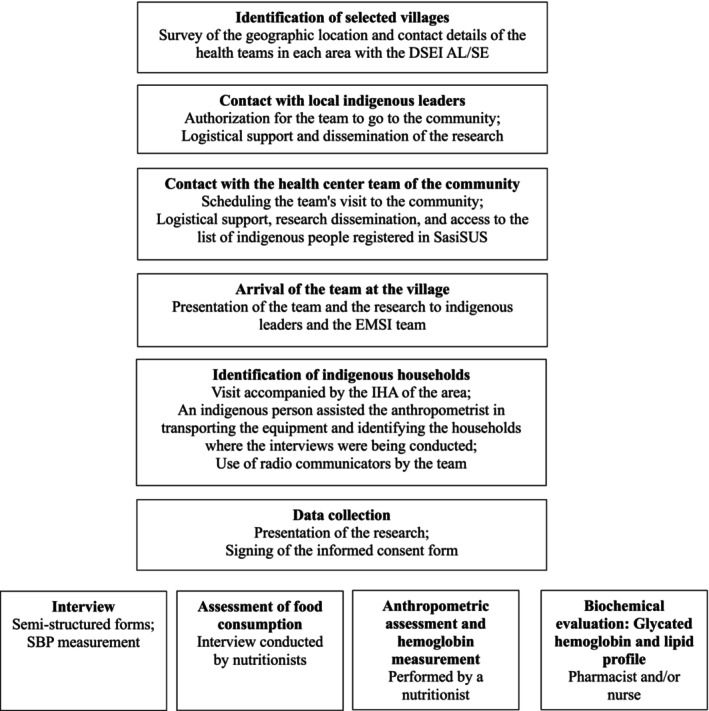
Flowchart of data collection. ENSSAIA, Alagoas, 2023. DSEI, Special Indigenous Health District of Alagoas and Sergipe; EMSI, Multidisciplinary Indigenous Health Team; IHA, Indigenous Health Agent; SasiSUS, Indigenous Health Care Subsystem; SBP, Systemic arterial pressure.

Figure 3 presents the data collection flowchart. To ensure effective team communication and track households with ongoing interviews, all team members used radio communicators.

The variables of interest were collected through household interviews based on predefined scripts, using electronic forms developed on the Epicollect5 platform, version 4.0.0, installed on Samsung tablets. Except for dietary intake data, all other forms were filled out directly within the system. After each data collection shift, the applied forms were uploaded.

At the end of each day, the field supervisor updated a specific Google spreadsheet for each community, containing all household identification and family registration data. An external researcher to the field team was responsible for downloading the spreadsheets generated by Epicollect and comparing these data with the information recorded in the control spreadsheet, verifying the application of forms according to the eligibility profile of each household participant. Identified inconsistencies were noted in the control spreadsheet. Based on these notifications, the field supervisor guided the interviewers on corrections, which could include returning to the household.

### 2.2 Team Training

The training began with the presentation and discussion of the overall project and proceeded in three phases: theoretical, practical, and a pilot study.

### 2.3 Interviewers

In the first phase (June 2022), the training included the presentation of the forms and the interviewer manual. In the second phase, interviewers were trained to apply the forms and use the equipment (June to August 2022), including interview simulations.

### 2.4 Anthropometrist

All anthropometric measurements and hemoglobin testing (for children ≥ 6 months to < 5 years and pregnant women) were conducted by a single anthropometrist, a trained nutritionist. Training included:

- Theoretical approach: lectures on anthropometric measurements and hemoglobin testing, reading the technical manual, and video demonstrations;
- Practical training: initially, height and length measurements for children under 5 years old were practiced in a preschool. Subsequently, other anthropometric measurements (weight, height, arm span, circumferences, and bioimpedance) and hemoglobin testing were trained among the team members.

To assess intra‐evaluator variability of measurements, each measurement was performed in duplicate by the anthropometrist and compared with those obtained by the instructors.

### 2.5 Laboratory Assistant

Hemoglobin A1c and lipid profile tests were performed by a pharmacist. The theoretical and practical training was provided by a biomedical professional experienced in using the equipment for field research and a company technician.

### 2.6 Pilot Study

The pilot study took place on August 30, 2022, with the objective of testing the adequacy of instruments and field logistics. The Wassú‐Cocal community was chosen for convenience due to its proximity to Maceió, facilitating planning and execution.

Following the pilot study, the produced materials were analyzed, and the forms underwent necessary adjustments to ensure greater precision and effectiveness in data collection.

### 2.7 Eligibility Criteria

Table 2 presents the definition of research participant eligibility and the types of assessments conducted according to this eligibility. The data collected in each household varied according to the profile of eligible residents, covering:

- Environmental, demographic, socioeconomic data, access to policies and public service infrastructure, and household food security;
- Nutritional status indicators: anthropometric, biochemical: hemoglobin (children 6 months to < 5 years and pregnant women), and dietary (women, pregnant women, and children < 5 years);
- Data on women's, children's, pregnant women's, and elderly health;
- Information on breastfeeding for children under 2 years old;
- Data on individuals with disabilities and genetic alterations;
- Information on noncommunicable diseases: diabetes mellitus (women and elderly), hypertension (women, men, and elderly), common mental disorders (women and men), cardiovascular diseases;
- Data on alcohol and tobacco use (women and men);
- Biochemical data: lipid profile and HbA1c (women);
- Genetic data: saliva collection for genetic polymorphism evaluation.

**TABLE 2:** Definition of the eligibility of each individual participating ENSSAIA, Alagoas, 2023.

Eligibility	Definition	Quantity assessed per household	Type of assessment carried out
Pregnant	Being pregnant, regardless of age	All pregnant women residing in the household	− Specific forms; − Anthropometric assessment; − Hemoglobin measurement
Children	Age range: 0–5 years, both sexes	All children residing in the household	− Specific forms; − Anthropometric assessment; − Hemoglobin measurement (children aged 6 months to < 5 years)
Schoolchildren	Age range: 5.1–10 years, both sexes	All schoolchildren residing in the household	− Family Registration; − Anthropometric assessment
Teenager	Age range: 10.1 to < 19 years, both sexes	All teenagers residing in the household	− Family Registration; − Anthropometric assessment
Eligible woman	Age range: 19–59 years	One woman per household, defined by simple draw	− Specific forms; − Anthropometric assessment; − Blood pressure measurement; − Biochemical assessment
Woman not eligible	Age range: 19 to 59 years	All other women residing in the household who were not selected	− Family Registration; − Anthropometric assessment
Mother of child	Woman of any age with child < 5 years old	All mothers of children	− Specific forms; − Anthropometric assessment; − Blood pressure measurement
Eligible man	Age range: 19 to 59 years	One man per household, defined by simple draw	− Specific forms; − Anthropometric assessment; − Blood pressure measurement
Man not eligible	Age range: 19–59 years	All other men residing in the household who were not selected	− Family Registration; − Anthropometric assessment
Elderly	Age range: ≥ 60 years, both sexes	All elderly people living in the household	− Specific forms; − Anthropometric assessment; − Blood pressure measurement
Person with disability	Both sexes, regardless of age	All people with disabilities residing in the household	− Specific forms
Person with genetic alteration	Both sexes, regardless of age	All people with genetic alterations in the household	− Specific forms

Thus, the data collected in each household varied according to the age group, sex, and physiological status of family members.

The forms used included protocols created by the research group, adapted from previous surveys, or validated instruments from other researchers (Table S2).

For the Household Identification, Demographic, Socioeconomic, and Environmental Characterization, and the Brazilian Food Insecurity Scale forms, interviews were conducted with the household head.

### 2.8 Data Collection Instruments

#### 2.8.1 Household Identification

This form included: municipality name; community and ethnicity name with respective identification codes; household identification number; interview date; interviewer name; and the number of household members; internet access in the community; and geographic location (GPS) of the home.

#### 2.8.2 Family Registration

The Family Registration form gathered data on all household members, including name, relationship to the household head, sex, age, eligibility, self‐reported race/ethnicity, employment status, monthly income, income source, COVID‐19 diagnosis, and diagnostic method. It also recorded estimated total household income, participation in government programs, and any COVID‐19‐related deaths in the family.

#### 2.8.3 Demographic, Socioeconomic, and Environmental Characterization

This section included: type of housing (predominant material); number of rooms; number of bedrooms; source and treatment of drinking water; waste disposal and sewage system; and the questions that compose the Brazilian Economic Classification Criteria, which classifies families in descending order of economic level (Associação Brasileira de Empresas de Pesquisa 2021).

#### 2.8.4 Brazilian Food Insecurity Scale (EBIA) Adapted for Indigenous Populations

Food security status (FS) was assessed using the Brazilian Food Insecurity Scale (EBIA), an instrument that allows for the direct measurement of household food security status. The process of adapting and validating the EBIA began in 2003, using the U.S. Household Food Security Survey Module (HFSSM) as a reference (Segall‐Corrêa et al. 2014). The results demonstrated high content and construct validity, predictive validity, and internal consistency, reinforcing the EBIA's reliability and external validity for assessing food insecurity in the Brazilian population (Segall‐Corrêa et al. 2004; Kepple and Segall‐Corrêa 2011). Specifically, studies have reported Cronbach's alpha values ranging from 0.80 to 0.93 across different populations, indicating high internal consistency (Pérez‐Escamilla et al. 2004; Melgar‐Quinonez et al. 2008). Additionally, the EBIA demonstrated construct validity through consistent associations with socioeconomic and dietary indicators (Instituto Brasileiro de Geografia e Estatística 2014) and high content validity due to its careful adaptation to the Brazilian context, ensuring cultural and conceptual appropriateness (Pérez‐Escamilla et al. 2004).

In 2004, this led to the official recommendation of the EBIA as a direct indicator for measuring household food security in national surveys. To date, it remains the primary instrument used to assess food insecurity in Brazil, contributing to the diagnosis of this condition and to the monitoring and evaluation of policies and programs aimed at combating hunger in the country (Segall‐Corrêa and Marin‐Leon 2009).

In 2014, the scale was refined and the current version has a 14‐question tool with yes/no responses about household food experiences over the past 3 months. Based on the number of affirmative answers, households were classified into levels ranging from concern about food scarcity to going an entire day without eating (Segall‐Corrêa et al. 2014).

Recognizing the specificities of Indigenous populations, Fávaro et al. (2007) proposed and validated in 2007 the EBIA‐G, an adapted version of EBIA for the Guarani population in São Paulo's coastal region. This version maintained 15 questions of the original version, adapting the language and shortening the reference period from 3 months to 1 month. The EBIA‐G demonstrated satisfactory internal consistency and psychometric qualities like those of the original EBIA, supporting the reliable use of both tools in household food insecurity assessments.

For this study, EBIA‐G was further adapted to align with the current 14‐question EBIA (excluding the weight loss question) while maintaining the three‐month reference period for comparability with other studies. Each affirmative response counted as 1 point. Households were classified based on total scores, ranging from 0 to 14 points for those with individuals under 18 and 0–8 points for those without (as the last six questions apply only to minors).

#### 2.8.5 Pregnant Women's Health

This form was given to all pregnant women in the household and included questions about: having a partner; intention to breastfeed; health problems in the 15 days before the interview; self‐reported diagnoses of diabetes, hypertension, and cardiovascular disease (before or during pregnancy); date of last menstruation; health issues during pregnancy; tobacco and alcohol use before and during pregnancy; medication use; and blood pressure measurement.

#### 2.8.6 Prenatal Care Assessment

The form was applied to all pregnant women and mothers of children ≤ 24 months. If there were multiple children in this age group, only the pregnancy of the youngest was assessed. Only women/mothers receiving prenatal care at the community health unit were included. Data collected covered prenatal care quality, ease of scheduling, average waiting time, presence of a companion during consultations, access to and support for medical exams, iron and folic acid supplementation, quality of medical and nursing care, participation in health education activities, and breastfeeding guidance. For mothers, additional questions covered final pregnancy weight and postpartum consultation attendance.

#### 2.8.7 Health of Children Under 5 Years

This form included: birth date, sex, prenatal care characterization, tobacco and alcohol use during pregnancy, pregnancy weight gain, type of delivery, birth condition, birth weight and length, anthropometric assessment records in the child's health booklet, immunization calendar updates, and presence of diarrhea, fever, and cough in the past 2 weeks, as well as hospitalizations in the last 12 months.

#### 2.8.8 Breastfeeding

The breastfeeding form was based on the “Nutrition and Health of Maternal and Infant Populations in Quilombola Communities of Alagoas” survey (Araújo et al. 2021), with adaptations incorporating additional questions from the “II Survey on Breastfeeding Prevalence in Brazilian Capitals and the Federal District” (Brasil. Ministério da Saúde 2009). The questions were directed at biological mothers of children under 2 years old.

#### 2.8.9 Evaluation of the National Iron Supplementation Program (PNSF) and the National Vitamin A Supplementation Program (PNSVA)

The PNSF form was applied to pregnant women and mothers of children aged 6–24 months. Questions assessed their knowledge of the program and its purpose, whether they received guidance on iron supplementation, understood its purpose, were using it (for pregnant women) or providing it to their child, its frequency and timing, participation in anemia‐related health education activities, and whether they had been diagnosed with anemia.

The PNSVA assessment targeted mothers of children aged 6–59 months, asking about program awareness, whether the child received supplementation, reasons for non‐receipt (e.g., unavailability at the health unit), and whether they were informed about vitamin A. The child's health booklet was also checked for supplementation records, including the number of doses and time since the last dose.

#### 2.8.10 Assessment of Women's Health

This form covered self‐reported race/ethnicity, marital status, whether the respondent was the head of the household, employment status, education level, age at menarche, contraceptive use, pregnancy history, number of pregnancies, live births, and deliveries, history of child loss, gynecological exams and surgeries, health issues in the last 15 days, previous chronic disease diagnoses, preferred healthcare service, current medication use, alcohol consumption the previous day, and blood pressure measurement.

#### 2.8.11 Self‐Report Questionnaire (SRQ‐20)

The mental health of women, mothers of children under 5 years old, and men was assessed using the Self‐Report Questionnaire (SRQ‐20), developed by the World Health Organization (WHO) and validated for the Brazilian population (Mari and Williams 1986). This instrument is designed for screening common mental disorders in epidemiological studies. It consists of 20 yes/no questions about emotional and physical symptoms associated with psychiatric conditions over the past 30 days, with each affirmative response corresponding to one point.

#### 2.8.12 Alcohol and Tobacco Screening Test

Alcohol and tobacco use among women and men was assessed using the Alcohol, Smoking, and Substance Involvement Screening Test (ASSIST), a validated tool for the Brazilian population (Henrique et al. 2004). It includes questions on the use of nine classes of psychoactive substances, but for this study, only alcohol and tobacco consumption were considered. The questions addressed lifetime and past three‐month use frequency, problems related to use, concern from friends or family, impairment in activities, attempts to quit or reduce use, and perceived dependence. Responses were scored from 0 to 4, with a total score (0–20) categorizing consumption as occasional use, abuse, or dependence (Henrique et al. 2004).

#### 2.8.13 Elderly Health and Vulnerable Elders Survey‐13 (VES‐13)

Health assessment for elderly participants was based on a general health questionnaire and the validated Vulnerable Elders Survey‐13 (VES‐13). The general health questionnaire covered education level, marital status, social support (living alone, presence of a companion/relative/friend, family assistance, availability of help when ill or incapacitated), history of fractures, fear of falling, number of falls in the past 3 months, presence of noncommunicable diseases, type of healthcare service used, smoking and alcohol consumption, medication use, and blood pressure measurement.

The VES‐13 is a screening tool for identifying vulnerable elderly individuals, covering 13 items related to self‐perceived health, physical limitations, and functional decline (Maia et al. 2012).

#### 2.8.14 Assessment of Individuals With Disabilities and Genetic Alterations

Forms evaluating individuals with disabilities or genetic alterations collected information on the type of disability/condition, need for government assistance, type of required assistance, and whether this assistance was being provided.

### 2.9 Dietary Intake Assessment

#### 2.9.1 24‐Hour Dietary Recall (24HR)

The 24‐h recall (24HR) was applied to eligible women, pregnant women, and mothers or caregivers responsible for feeding children under five. This dietary assessment method identifies and quantifies food and beverage intake from the day before the interview, including details about ingredients, preparation methods, portion sizes in household measures, and meal timing. Dietary intake data were collected using a single 24HR. To reduce memory bias and assist with portion quantification, two photographic food albums were used (Monteiro et al. 2007; Crispim et al. 2017).

Interviews followed the three‐step multiple‐pass method recommended by the United States Department of Agriculture (USDA) (Johnson et al. 1998). One of the nutritionists, with extensive experience in applying this method in population‐based studies, trained the other interviewer.

#### 2.9.2 Dietary Intake: Vigitel

Questions from the Surveillance System for Risk and Protective Factors for Chronic Diseases by Telephone Survey (Vigitel) were applied to eligible women and pregnant women to assess food and beverage consumption. These questions covered: (1) frequency of consumption of beans, raw and cooked vegetables, natural fruit juice, fruits, soda, or artificial juice; (2) type of soda consumed and daily intake quantity (in glasses); (3) a list of some unprocessed and minimally processed foods to assess consumption on the previous day; and (4) a list of some ultra‐processed foods to assess consumption on the previous day. The details of these questions are available in another publication (Brasil 2022).

#### 2.9.3 Markers of Food Consumption From the Ministry of Health

An adapted version of the Food Consumption Markers Form from the Food and Nutrition Surveillance System was applied to mothers or caregivers of children under 24 months old (Brasil 2015). This form identifies healthy and unhealthy dietary practices through specific questions for different age groups. For this study, only questions related to food consumption on the previous day were used for: (1) children under 6 months old (assessing early introduction of foods); and (2) children aged six to 23 months and 29 days (assessing consumption of foods protective against or associated with micronutrient deficiencies and overweight risk). The response options were “yes,” “no,” or “don't know.”

#### 2.9.4 Tea Consumption

The form was administered to mothers of children aged ≤ 24 months who were currently being breastfed or had been breastfed. The questions included whether tea had ever been offered and, if so: the type of tea (herb name), frequency and volume of consumption, purpose of use (therapeutic, hydration, or nutritional), who provided guidance on its use, and where the herb was obtained.

#### 2.9.5 Anthropometric Assessment

Table S3, describes the anthropometric measurements taken and the equipment used, according to participant eligibility. Only length measurements were taken in duplicate, involving three individuals: the anthropometrist, the field supervisor, and the child's mother or caregiver.

To ensure accurate data recording, the anthropometrist verbally announced each measurement result, which was repeated aloud by the interviewer for confirmation before immediate entry into the form.

#### 2.9.6 Blood Pressure Measurement

Blood pressure was measured three times, with the participant (eligible woman/man, pregnant woman, and elderly individual) seated and at rest for 15 min before measurement. Omron digital devices (model HEM‐742, São Paulo, Brazil) were used. Measurements followed the recommendations of the 2020 Brazilian Hypertension Guidelines (Barroso et al. 2021).

This procedure was performed for eligible women and men, all elderly participants, and pregnant women in the household.

#### 2.9.7 Biochemical Assessment

Hemoglobin levels were measured in pregnant women and children aged ≥ 6 months to < 5 years. Hemoglobin A1c and lipid profile (total cholesterol, triglycerides, HDL cholesterol, and LDL cholesterol) were assessed in eligible women. Due to the operational impracticality of fasting for hemoglobin A1c and lipid profile testing, current recommendations for non‐fasting biochemical exams were followed (Faludi et al. 2017; Cobas et al. 2022).

For hemoglobin measurement, a drop of blood was obtained through a fingerstick from the child or pregnant woman. The determination was performed using a portable photometer (Compolab TM, Fresenius Kabi, São Paulo, Brazil) with specific cuvettes for the equipment. The result was recorded in the Family Registration and Nutritional Status Assessment form and included in a printed report provided to the mother or child's caregiver and the IHA.

For hemoglobin A1c and lipid profile measurements, automatic disposable lancets were used to obtain 20 μL of capillary blood via fingerstick from eligible women. The samples were stored in microtubes with EDTA in an insulated container with gel packs and analyzed using a portable multi‐assay analyzer (Afinion 2, Abbott, USA) with respective test cartridges. Blood was transferred from microtubes to the test cartridges using automatic pipettes. The hemoglobin A1c cartridge required 1.5 μL of blood, with a reading time of approximately 3 min, while the lipid profile cartridge required 15 μL and approximately 7 min for analysis. Results were printed and affixed to a record book, exported to a USB drive, and later saved on the research laboratory computer. They were also transcribed into reports delivered by the IHA to the participant. If abnormal results were detected, the laboratory assistant informed the IHA, who scheduled a medical consultation for the participant.

#### 2.9.8 Genetic Polymorphism Analysis

Oral mucosa cell samples were collected from eligible participants for genetic polymorphism analysis. Before collection, they rinsed their mouths with 10 mL of saline. Samples were obtained by scraping the inner cheeks with sterile cytology brushes in about 30 circular motions. The brush tips were then cut and stored in 2 mL microtubes with 0.9% saline. Samples were kept in insulated containers during fieldwork, refrigerated for transport to UFAL, and stored at −20°C until analysis.

#### 2.9.9 Data Consistency Analysis

Researchers from the University of São Paulo (USP) managed and verified weekly databases (CSV spreadsheets) for each eligible group throughout data collection. Databases were downloaded from Epicollect and stored securely on Google Drive. This verification ensured data quality by detecting inconsistencies, missing information, or errors early, allowing corrections while the team was still in each community.

After the initial review, databases were imported into Stata 18.0 (StataCorp, TX, USA), merged by eligible groups, and structured into files containing household and participant data. As new communities were surveyed, the process was repeated. Verification do‐files were created using statistical software and original forms to ensure data reliability and accuracy.

Data analysis will include both descriptive and inferential statistics, depending on the nature of the variables and their adherence to the assumptions of parametric tests. Identification of associated factors and control for potential confounders will be conducted using multivariable regression models.

## 3 Discussion

ENSSAIA was a pioneering initiative that comprehensively addressed the health, nutrition, and food security conditions of the Indigenous population in Alagoas, filling a significant gap in the epidemiological landscape at both the state and national levels.

Until then, studies conducted in Alagoas had focused only on specific ethnic groups, without comprehensively covering the diversity of Indigenous peoples in the state (Pereira et al. 2012; Campos et al. 2016). At the national level, only one survey was conducted between 2008 and 2009, aiming to describe the food and nutritional situation and its determining factors among Indigenous children under 5 years of age and Indigenous women aged 14–49 years. This survey used a probabilistic sample of 113 villages distributed across the four major regions of the country (North, Northeast, Central‐West, and Southeast/South), ensuring both national and regional representativeness. However, it was not possible to disaggregate the data by state, and in the case of Alagoas, only three villages were included (Cardoso et al. 2009).

It is important to highlight that this initiative took place decades later compared to the non‐Indigenous population, for whom national surveys on health, nutrition, and food security have been conducted since the 1970s without including Indigenous peoples.

Neither the local studies nor the national survey encompassed the diversity of objectives and eligible groups addressed by ENSSAIA. The methodology was carefully designed to ensure representativeness, statistical robustness, and cultural sensitivity, covering all stages from sampling design to data collection and validation procedures.

The sampling design notably incorporated strategies aimed at ensuring the production of robust estimates for the outcomes assessed (Lwanga et al. 1991; Kotrlik and Higgins 2001) as well as the use of methods widely established in national and international research, such as:

- Application of the 24HR for assessing food consumption (Food and Agriculture Organization of the United Nations 2018), which will allow: (1) the analysis of dietary patterns using a posteriori methods, considering the effects of multiple food groups on health (Newby and Tucker 2004); and (2) the classification of foods according to the NOVA framework, an internationally recognized approach widely used in epidemiological studies to assess food processing levels (Monteiro et al. 2019);
- Use of questions on food consumption from the previous day, adopted since 2018 by the Vigitel system of the Brazilian Ministry of Health, which annually monitors risk and protective factors for noncommunicable diseases (NCDs) in Brazilian state capitals (Brasil 2019);
- Assessment of breastfeeding practices, enabling the estimation of indicators recommended by the WHO (World Health Organization 2008) and endorsed by the Brazilian Ministry of Health (Brasil. Ministério da Saúde 2009);
- Assessment of nutritional status using anthropometric indicators widely recognized and employed in epidemiological studies (World Health Organization 2019, 1995);
- Identification of chronic diseases based on national guidelines, including blood pressure measurement for the diagnosis of systemic arterial hypertension (Barroso et al. 2021) and glycated hemoglobin (HbA1c) testing for the diagnosis of diabetes *mellitus* (Cobas et al. 2022);
- Collection of biological material for the investigation of genetic polymorphisms, employing a standardized DNA extraction technique from oral mucosa cell swabs using saline solution (sodium chloride) (Abrão et al. 2005) in a population that is underrepresented in genetic studies;
- Use of instruments validated for the Brazilian population, some adapted from international versions, such as the SRQ‐20 (Mari and Williams 1986); ASSIST (Henrique et al. 2004); VES‐13 (Maia et al. 2012); and the EBIA (Segall‐Corrêa et al. 2014; Fávaro et al. 2007) (adapted version for Indigenous peoples), the main tool for assessing food insecurity in Brazil since 2004.

The adoption of these methods enables not only the estimation of priority conditions for the public policy agenda but also the comparison of results with other national and international studies involving both Indigenous and non‐Indigenous populations.

The data collection process highlighted the crucial role of collaboration between researchers and indigenous leaders in ensuring ethical and culturally sensitive execution. Comprehensive field team training and continuous consistency checks strengthened data reliability and quality. These procedures minimized the occurrence of random and systematic errors in measurements and database construction, based on well‐established references in the literature (Szklo and Nieto 2014) and the accumulated experience of the involved research groups.

The results generated by ENSSAIA will make a significant contribution to filling knowledge gaps regarding the magnitude and factors associated with nutrition, health, and food security conditions in Indigenous villages in Alagoas. The evidence produced may support the formulation, adjustment, and monitoring of public policies aligned with the specific needs of this population and other groups with similar socioeconomic and cultural characteristics, while respecting local traditions and knowledge. Additionally, the relevance of these findings for priority issues on the national public health agenda is underscored.

Furthermore, in Brazil, there are legal provisions that ensure community participation in the management of the SUS (Unified Health System), aiming to strengthen social control in the formulation of strategies and the monitoring of the implementation of public health policies at various levels of the system. This mechanism seeks to guarantee transparency, efficiency, and quality of services provided, as well as promote the democratization of SUS management. In this context, returning the results to the communities participating in ENSSAIA represents not only an ethical commitment to transparency and feedback of the findings but also a tool to strengthen the link between research and social action. By fostering this connection, it directly supports organized civil society, providing subsidies for qualified social control and advocacy for culturally appropriate, equitable, and evidence‐based public health policies (Brasil. Presidência da República 1990; BRASIL 2025).

Specifically, the findings may directly impact policies, programs, and health services aimed at Indigenous peoples, with the potential to promote food and nutritional security; encourage, protect, and support breastfeeding; prevent and control NCDs and their risk factors; combat various forms of malnutrition; and foster health promotion and quality of life, especially among priority groups such as pregnant women, children, and the elderly.

Additionally, the study will establish a baseline for understanding the epidemiological patterns of the conditions evaluated—being the first survey to include all Indigenous ethnic groups in the state. It is expected that this study will serve as a methodological model for future research, which may deepen the understanding of the inequalities faced by Indigenous populations in different contexts, contributing to the development of a more equitable, inclusive, and culturally sensitive public health landscape in Brazil.

In this regard, it is important to highlight that conducting this study involved numerous challenges that should be carefully considered when replicating the research among Indigenous groups in other regions, whether in Brazil or elsewhere. It is also noteworthy that many of these challenges are inherent to population‐based surveys, regardless of whether they focus on Indigenous peoples. With the aim of supporting the implementation of future studies of this nature, the main points that require attention are listed below:

- Adaptation of data collection instruments: Given the ethnic diversity, it may be necessary to adapt the instruments, especially regarding language and validity for the target group. Ideally, the project design should involve representatives of the Indigenous groups, which facilitates the cultural appropriateness of both the instruments and the fieldwork logistics according to local realities;
- Logistical challenges of fieldwork: The complexity of fieldwork in Indigenous territories—characterized by the dispersion of villages, the long distances between households (many with difficult access), and demographic fluctuations—requires effective coordination with key stakeholders, such as community leaders and health teams. This engagement is essential both for raising awareness among families and for ensuring the dissemination and acceptance of the research within the communities;
- Communication with villages and access permissions: In Brazil, access to Indigenous villages requires prior negotiation with local leaders, such as chiefs or shamans, followed by engagement with health teams. This step must be preceded by presenting the research, as was done in this study, both during the project development and before and during data collection. Due to the COVID‐19 pandemic and changes in local leadership, it was necessary to re‐present the project, highlighting the importance of preparatory stages, as outlined in Flowchart 1.
- Diversity and involvement of Indigenous people in data collection: Brazil has one of the greatest Indigenous diversities in the Americas, with around 200 officially recognized groups speaking approximately 180 languages. These groups have varied experiences of interaction with broader society. Therefore, it is essential to include Indigenous individuals as part of the fieldwork team. In contexts with language barriers—which was not the case in this study—the presence of translators becomes indispensable.
- Sensitivity to cultural dynamics: Research on the health and nutrition of Indigenous peoples must consider not only epidemiological and demographic aspects but also demonstrate respect for local traditions and customs. This includes adapting the research schedule to the village's activities and rituals, which often involve the entire community, making data collection impossible during those periods. This underscores, once again, the importance of continuous coordination between researchers and local leaders.

## 4 Conclusion

ENSSAIA stands as a pioneering and comprehensive initiative that fills a critical gap in the epidemiological knowledge about the nutrition, health, and food security of Indigenous peoples in the state of Alagoas, Brazil. The methodological rigor adopted—from sampling design to data collection and quality control—ensures that the evidence produced is both reliable and representative of the studied population. By addressing all Indigenous ethnic groups in the state and incorporating a wide range of health, nutrition, and social indicators, the study not only contributes to the formulation and improvement of public policies but also serves as a methodological benchmark for future research involving Indigenous populations or other socially vulnerable groups. Furthermore, the detailed description of the methods used is intended to guide other researchers interested in conducting similar studies, favoring the adoption of comparable methodologies that strengthen the consistency and comparability of findings across different contexts. Moreover, the experience accumulated highlights the importance of community participation, cultural sensitivity, and ethical commitment in conducting research within Indigenous contexts. It is expected that ENSSAIA will not only inform local actions but also stimulate broader discussions on equity, health, and human rights, contributing to the advancement of more inclusive and culturally respectful public health strategies in Brazil and beyond.

## Author Contributions

T.R.S., M.A.C., H.S.F., A.H.S., E.A.S., L.B.B., M.L.A., W.H.C.M., and R.C.S.V. were responsible for drafting the project. T.R.S., H.S.F., and A.H.S. coordinated the training of the data collection team. T.R.S., H.S.F., A.H.S., and W.H.C.M. participated in field data collection. V.C.O. and M.A.C. were responsible for data consistency analysis throughout the collection process. T.R.S., H.S.F., M.A.C., and V.C.O. drafted the initial version of the manuscript. All authors critically reviewed the manuscript and approved the final version.

## Funding

This project received financial support from the National Council for Scientific and Technological Development (CNPq; grant no. 432249/2018‐4), the Alagoas State Research Support Foundation (FAPEAL; grant no. 600300000000460/2020), the São Paulo State Research Support Foundation (FAPESP; grant no. 2019/22739‐4), and the Coordination for the Improvement of Higher Education Personnel (CAPES; grant no. 88887.836261/2023‐00). TRS received doctoral and postdoctoral scholarships from FAPEAL (grants no. 88887.227722/2018‐00 and 88887.809525/2023‐00, respectively). VCO received a master's scholarship from CAPES—Brazil (PROEX, grant no. 88887.663687/2022‐00). MAC (grant no. 303794/2021‐6) and HSF (grant no. 313991/2021‐9) are research productivity fellows of CNPq.

## Ethics Statement

The project was submitted to the Indigenous Health District Council (CONDISI) and, after approval, was reviewed and approved by the UFAL Research Ethics Committee and the National Research Ethics Commission (CONEP; CAAE: 29121120.0.0000.5013). Researchers were only allowed to enter indigenous territories following approval from local leaders (chief and spiritual leader). Participation in the research was contingent upon the participant's consent through the signing of an informed consent form.

## Conflicts of Interest

The authors declare no conflicts of interest.

## Supporting information


**Table S1:** Distribution of Indigenous communities in Alagoas by region, city, and ethnicity. Study of Nutrition, Health, and Food Security of the Indigenous Peoples of Alagoas (ENSSAIA), 2023.
**Table S2:** Forms used and the respective eligible groups for each. ENSSAIA, Alagoas, 2023.
**Table S3:** Description of the anthropometric measurements, according to the eligibility of each participant. ENSSAIA, Alagoas, 2023.

## Data Availability

The datasets used and/or analyzed during the current study are available from the corresponding author on reasonable request.
