# Supplementary material for: Study of Nutrition, Health, and Food Security of Indigenous Peoples in the State of Alagoas, Brazil (ENSSAIA): Methodological Aspects
Source: Food Sci Nutr. 2025 Dec 25;13(12):e71361. doi: 10.1002/fsn3.71361 (PMC12741332; doi:10.1002/fsn3.71361)
Supplement: Supplementary file 1 — Table S1: Distribution of Indigenous communities in Alagoas by region, city, and ethnicity. Study of Nutrition, Health, and Food Security of the Indigenous Peoples of Alagoas (ENSSAIA), 2023. Table S2: Forms used and the respective eligible groups for each. ENSSAIA, Alagoas, 2023. Table S3: Description of the anthropometric measurements, according to the eligibility of each participant. ENSSAIA, Alagoas, 2023. [file FSN3-13-e71361-s001.docx]

**Table 1 (Supplementary Material).** Distribution of Indigenous communities in Alagoas by region, city, and ethnicity. Study of Nutrition, Health, and Food Security of the Indigenous Peoples of Alagoas (ENSSAIA), 2023.

| **Region** | **City** | **Ethnicity** | **Community** |
| --- | --- | --- | --- |
| Agreste | Traipu | Aconã | Aconã |
|  | São Sebastião | Karapotó | Fazenda Terra Nova |
|  |  |  | Plaki-ô |
|  | Campo Grande, Feira Grande | Tingui-Botó | Tingui-Botó |
|  |  |  | Olho D’Água do Meio |
| Alto Sertão | Água Branca | Kalankó | Januária |
|  |  |  | Lajedo do Couro |
|  |  |  | Sítio Gregório |
|  | Inhapi | Koiunpanká | Baixa do Galo |
|  |  |  | Roçado |
|  |  |  | Baixa Fresca |
|  | Pariconha | Geripankó | Ouricuri |
|  |  |  | Figueiredo |
|  |  |  | Moxotó |
|  |  |  | Serra do Engenho |
|  |  |  | Aratikun |
|  | Pariconha | Karuazu | Tanque |
|  |  |  | Campinhos |
|  | Pariconha | Katokinn | Katokinn |
| Baixo São Francisco | São Brás, Porto Real do Colégio | Kariri-Xokó | Kariri-Xokó |
| Planalto da Borborema | Palmeira dos Índios | Xucuru-Kariri | Fazenda do Canto |
|  |  |  | Boqueirão |
|  |  |  | Mata da Cafuna |
|  |  |  | Cafurna de Baixo |
|  |  |  | Serra da Capela |
|  |  |  | Serra do Amaro |
|  |  |  | Coité |
|  |  |  | Riacho Fundo |
| Serra dos Quilombos | Colônia Leopoldina, Joaquim Gomes, Matriz de Camaragibe, Novo Lino | Wassú | Wassú-Cocal |
| **TOTAL** | | **11** | **29** |

**Source:** Secretaria de Estado do Planejamento, Gestão e Patrimônio (Secretaria de Estado do Planejamento Gestão e Patrimônio, 2017).

**Table 2 (Supplementary Material).** Forms used and the respective eligible groups for each. ENSSAIA, Alagoas, 2023.

| **Forms** | **Form Type*** | **Eligible group** |
| --- | --- | --- |
| Household Identification | Own form | Famíly |
| Family Registration | Own form | Famíly |
| Demographic, Socioeconomic, and Environmental Characterization | Own form | Famíly |
| Brazilian Food Insecurity Scale (EBIA) Adapted for Indigenous Populations | Validated form ¹ | Famíly |
| Pregnant Women’s Health | Own form | Pregnant |
| Prenatal Care Assessment | Own form | Pregnant  Children ≤ 24 months |
| Health of Children Under 5 Years | Own form | Children < 5 years old |
| Breastfeeding | Own form | Children ≤ 24 months |
| Evaluation of the National Iron Supplementation Program (PNSF) | Own form | Children de 6 to ≤ 24 months |
| Evaluation of the National A Supplementation Program (PNSVA) | Own form | Children < 5 years old |
| Assessment of Women’s Health | Own form | Eligible woman (19-59 years old) |
| Self Report Questionnaire (SRQ-20) | Validated form^2^ | Eligible woman (19-59 years old)  Eligible man (19-59 years old) |
| Alcohol and Tobacco Screening Test | Validated form^3^ |  |
| Elderly Health | Own form | Elderly – both sexes (≥ 60 years old) |
| Vulnerable Elders Survey-13 (VES-13) | Validated for ^4^ | Elderly – both sexes (≥ 60 years old) |
| Assessment of Individuals with Disabilities | Own form | Person with disability |
| Assessment of Individuals with Genetic Alterations | Own form | Person with genetic alteration |
| Dietary Intake Assessment: 24-Hour Dietary Recall (24HR) | Own form | Children < 5 years old  Eligible woman (19-59 years old) Pregnant |
| Dietary Intake Assessment: Vigitel | Adapted form^5^ | Eligible woman (19-59 years old) Pregnant |
| Dietary Intake Assessment: Markers of Food Consumption from the Ministry of Health | Adapted form^6^ | Children ≤ 24 months |
| Dietary Intake Assessment: Tea Consumption | Own form | Children ≤ 24 months |
| Anthropometric Assessment | Own form | Family |
| Biochemical Assessment: hemoglobin | Own form | Children de 6 months to < 5 years old  Pregnant |
| Biochemical Assessment: hemoglobin A1c and lipid profile | Own form | Eligible woman (19-59 years old) |

*The own forms were prepared by the research group, based on the adaptation of forms prepared for previous research: ^1^(Fávaro, Ribas, Zorzatto, Segall-Corrêa, & Panigassi, 2007); ^2^(Mari & Williams, 1986); ^3^(Henrique, De Micheli, Lacerda, Lacerda, & Formigoni, 2004); ^4^(Maia, Duarte, Secoli, Santos, & Lebrão, 2012); ^5^(Brasil, 2022); ^6^(Brasil, 2015).

**Table 3 (Supplementary Material).** Description of the anthropometric measurements, according to the eligibility of each participant. ENSSAIA, Alagoas, 2023.

| **Measures** | **Equipment** | **Eligible for the measure** | **Responsible for the measures** | **Description of measures** |
| --- | --- | --- | --- | --- |
| Weight (kg) | Portable digital scales (Seca, model 813), with a capacity of 200 kg and 100g subdivisions | All family members, except for people with physical disabilities | Anthropometrist | The participant stood barefoot in the center of the platform, distributing their weight evenly on the equipment, with a straight posture, head in the anatomical position, and arms extended along the body, removing objects from their pockets and coats |
| Weight (kg) of children <2 years | Portable digital scales (Seca, model 813), with a capacity of 200 kg and 100g subdivisions | Children < 2 years old | - Anthropometrist  - Mother or guardian of the child | The weight of the child's mother/guardian was measured (as described in the weight measurement procedure). Then, the mother/guardian was weighed while holding the child, and the child's body mass was obtained by subtracting the first measurement from the second |
| Length (cm) | Pediatric stadiometer (Seca®, model 417), with a measuring range of 10 to 100 cm and subdivisions in mm | Children < 2 years old | - Anthropometrist  - Field Supervisor  - Mother or guardian of the child | The child was measured barefoot, wearing minimal clothing, and without any objects on the head. The stadiometer was placed on a flat surface, and the mother or guardian positioned the child supine on the equipment, with shoulders and back in contact with the surface. The arms were either alongside the body or held over the abdomen by the mother or guardian. The supervisor ensured that the child’s eyes were aligned with the Frankfurt plane, neck straight, and chin away from the chest. The anthropometrist held the legs, gently pressing the knees to keep them straight. Once the supervisor confirmed the correct positioning, the anthropometrist moved the footplate to the soles of the feet, forming a right angle with the legs, and recorded the measurement |
| Height (cm) | Portable vertical stadiometer (Seca®, model 213), with range up to 205 cm and subdivisions in mm | All family members, except for people with physical disabilities and children < 2 years old | - Anthropometrist  - Field Supervisor  - Interviewer (children 2-5 years old) | The measurement was taken with individuals in an upright position. They were asked to remove shoes, socks, and any objects from their heads. Everyone rested their head, back, buttocks, and heels against the vertical rod of the stadiometer, with arms relaxed at their sides, palms facing their thighs, and their eyes at a 90º angle (Frankfurt plane). The heels were together, and the feet were positioned at an approximately 60º angle. The anthropometrist, standing laterally, slid the horizontal rod of the highest point of the head, forming a 90º angle with the vertical rod, and applying enough pressure to compress the hair. The reading was taken at the anthropometrist's eye level. For children aged 2 to 5 years, the interviewer or field supervisor assisted in maintain the correct position |
| Arm span | Vertical rod of the portable vertical stadiometer (Seca®, model 213), with amplitude up to 205 cm and subdivisions in mm | All elderly people in the household | - Anthropometrist  - Interviewer | The elderly participant was instructed to remove any clothing that could hinder arm extend, stand up and lean against the wall. With weight evenly distributed between both feet and heels against the wall, they extended their arms laterally at shoulder height (forming a 90º angle to the torso), keeping elbows straight, palms facing forward, and fingers together. To record the measurement, the interviewer aligned the zero mark of the inelastic tape scale with the distal phalanx of the middle finger on one hand, while the anthropometrist took the reading at the other end |
| Electrical bioimpedance (%) | Bipolar bioimpedance Omron HBF-306 (Omron Healthcare, Kyoto, Japan | - All teenagers;  - Eligible women;  - Eligible men;  - All elderly people | Anthropometrist | The anthropometrist entered the subject's data (weight, height, age, sex, and athletic level). The subject removed any metal objects in contact with the skin, stood with feet slightly apart, and placed both hands on the electrodes of the monitor strap. The palms wrapped around the top and bottom of the electrodes; with the middle finger position in the groove of the strap and the thumb resting on the top of the monitor. The arms were kept straight at a 90º angle to the body. The anthropometrist then pressed the START button, initing the measurement. After a few seconds, the result appeared on the display |
| Head circumference (cm) | Inelastic tape, with sensitivity to 0.1 mm (Cescorf®);  For children <5 years: measuring tapes for head circumference, with a capacity of 59 cm and accuracy of 0.1 mm (Seca, model 212) | - All children < 5 years old;  - All pregnant women;  - Eligible women | Anthropometrist | To perform the measurement, the fronto-occipital perimeter was considered. The measuring tape was wrapped around the head at its widest circumference, just above the eyebrows, passing over the supra-auricular line (above the ears) and around the occipital prominence (the most prominent part of the back of the head) |
| Neck circumference (cm) | Inelastic tape, with sensitivity to 0.1 mm (Cescorf®) | - All pregnant women;  - Eligible woman | Anthropometrist | The measurement was taken at the midpoint of the neck, at the level of the cricoid cartilage, between the middle of the cervical spine and the middle of the anterior neck. The woman stood in an orthostatic position, with her head in the Frankfurt plane, shoulders relaxed, and arms at her sides. The anthropometrist positioned the measuring tape firmly, applying minimal pressure to avoid compressing the skin, and recorded the measurement within five seconds to minimize discomfort |
| Waist circunference (cm) | Inelastic tape, with sensitivity to 0.1 mm (Cescorf®) | - Eligible woman;  - Eligible man;  - All elderly people | Anthropometrist | The measurement was taken at the level narrowest abdominal circumference (anatomical waist) or at the midpoint between the last rib and the iliac crest, if the anatomical waist was not identifiable. The participant stood upright with arms at the side of the body, feet close together, and weigth evenly distributed over both legs. Clothing was adjusted to expose the waist area. The anthropometrist instructed the participant to relax and breathe naturally, stood in front of them, identified the narrowest point or midpoint, wrapped the tape around the region, ensuring it was parallel to the floor, and took the measurement at the end of a normal exhalation. The tape was adjusted comfortably over the skin without compressing it. To maintain privacy, the anthropometrist took the reading while positioned next to the participant |

**REFERENCES**

Brasil. (2015). *Orientações para avaliação de marcadores de consumo alimentar na atenção básica*. In (pp. 33). Retrieved from https://bvsms.saude.gov.br/bvs/publicacoes/marcadores_consumo_alimentar_atencao_basica.pdf

Brasil. (2022). *Vigitel Brazil 2006-2021: Surveillance of risk and protective factors for chronic diseases through telephone surveys: Estimates on frequency and sociodemographic distribution of nutritional status and food consumption in the capitals of Brazil’s 26 states [Vigitel Brasil 2006-2021: vigilância de fatores de risco e proteção para doenças crônicas por inquérito telefônico: estimativas sobre frequência e distribuição sociodemográfica do estado nutricional e consumo alimentar nas capitais dos 26 estados brasileiros]*. In. Retrieved from http://bvsms.saude.gov.br/bvs/publicacoes/

Fávaro, T., Ribas, D. L., Zorzatto, J. R., Segall-Corrêa, A. M., & Panigassi, G. (2007). [Food security in Teréna indigenous families, Mato Grosso do Sul, Brazil]. *Cad Saude Publica, 23*(4), 785-793. Retrieved from https://www.scielo.br/j/csp/a/HrQfQRV38LBKB3KvVkNgNxk/?lang=pt doi:10.1590/s0102-311x2007000400006

Henrique, I. F. S., De Micheli, D., Lacerda, R. B. d., Lacerda, L. A. d., & Formigoni, M. L. O. d. S. (2004). Validação da versão brasileira do teste de triagem do envolvimento com álcool, cigarro e outras substâncias (ASSIST). *Revista da Associação Médica Brasileira, 50*, 199-206. Retrieved from https://www.scielo.br/j/ramb/a/TkCS3f3b5Nrm49tYRxW45Dm/?format=pdf&lang=pt

Maia, F. d. O. M., Duarte, Y. A. d. O., Secoli, S. R., Santos, J. L. F., & Lebrão, M. L. (2012). Cross-cultural adaptation of the Vulnerable Elders Survey-13 (VES-13): helping in the identification of vulnerable older people. *Revista da Escola de Enfermagem da USP, 46*, 116-122. Retrieved from https://www.scielo.br/j/reeusp/a/rFRhbJC69dGGmvYcZyj55JR/?format=html&lang=en

Mari, J. J., & Williams, P. (1986). A validity study of a psychiatric screening questionnaire (SRQ-20) in primary care in the city of Sao Paulo. *Br J Psychiatry, 148*, 23-26. Retrieved from https://www.cambridge.org/core/product/identifier/S0007125000209264/type/journal_article doi:10.1192/bjp.148.1.23

Secretaria de Estado do Planejamento Gestão e Patrimônio. (2017). *Estudo sobre as Comunidades Indígenas de Alagoas*. In. Retrieved from https://dados.al.gov.br/catalogo/dataset/176ce785-d4fd-47e4-b050-79e2e2aec220/resource/31957252-b75b-41fc-886d-4eab80504b2a/download/indigena.pdf
